# AlcoLab: a method for methanol screening using density and syringe-flow measurements, implemented as an open-source application

**DOI:** 10.1016/j.mex.2026.104211

**Published:** 2026-10-19

**Authors:** Diego Mendes de Souza, Pedro Augusto de Oliveira Morais, Nayara Ferreira Santos, Romério Rodrigues dos Santos Silva

**Affiliations:** a Baru Tech, Brasília, DF, Brazil; b Universidade Federal do Maranhão (UFMA), Centro de Ciências Exatas e Tecnologia (CCET), Campus Bacanga, São Luís, MA, Brazil; c Universidade Federal do Maranhão (UFMA), Centro de Ciências de Chapadinha, Chapadinha, MA, Brazil

**Keywords:** Methanol screening, Alcoholic beverage adulteration, Low-cost analytical method, Syringe-flow viscometry, Open-source progressive web app

## Abstract

Methanol adulteration in alcoholic beverages remains a recurrent public health problem. Instrumental methods for detecting methanol are not always available in field settings, while colorimetric methods often require toxic or hazardous reagents. Here we present AlcoLab, a proof-of-concept method for semiquantitative methanol screening in hydroalcoholic mixtures using a balance readable to 0.1 g and a 20-mL disposable syringe fitted with a 22-gauge needle. A ternary viscosity grid was fitted and selected using literature data (46-point model-selection benchmark: MAE ≈ 0.043 mPa·s; MAPE ≈ 2.47%), together with an empirical model linking syringe-flow response to viscosity. Under conditional blocked evaluation over 54 eligible replicates — including 30 measurements in commercial distilled spirits and commercial ethanol, 14 of which were methanol-spiked — the model yielded a MAE of 0.026 for the methanol mass fraction and flagged all 26 methanol-containing replicates, at a 26% false-positive rate over 84 negative predictions. Because the inversion was constrained to a ±2 percentage-point window around the nominal total alcohol content, these figures characterize the flow and inversion stages; all positives contained more than 16% methanol by mass, so performance at low methanol fractions remains to be established. The method is distributed as an open-source progressive web app (alcolab.org; AGPL-3.0) for methanol screening in suspected samples of distilled spirits and commercial ethanol solutions.

• Reagent-free methanol screening by density, syringe-flow measurements, and ternary inversion.
• Open-source AlcoLab application, with statistical interpretation and traffic-light classification.
• Reproducible workflow with a ternary grid, rerunnable scripts, and supplementary videos.

## Graphical abstract

**Figure:**
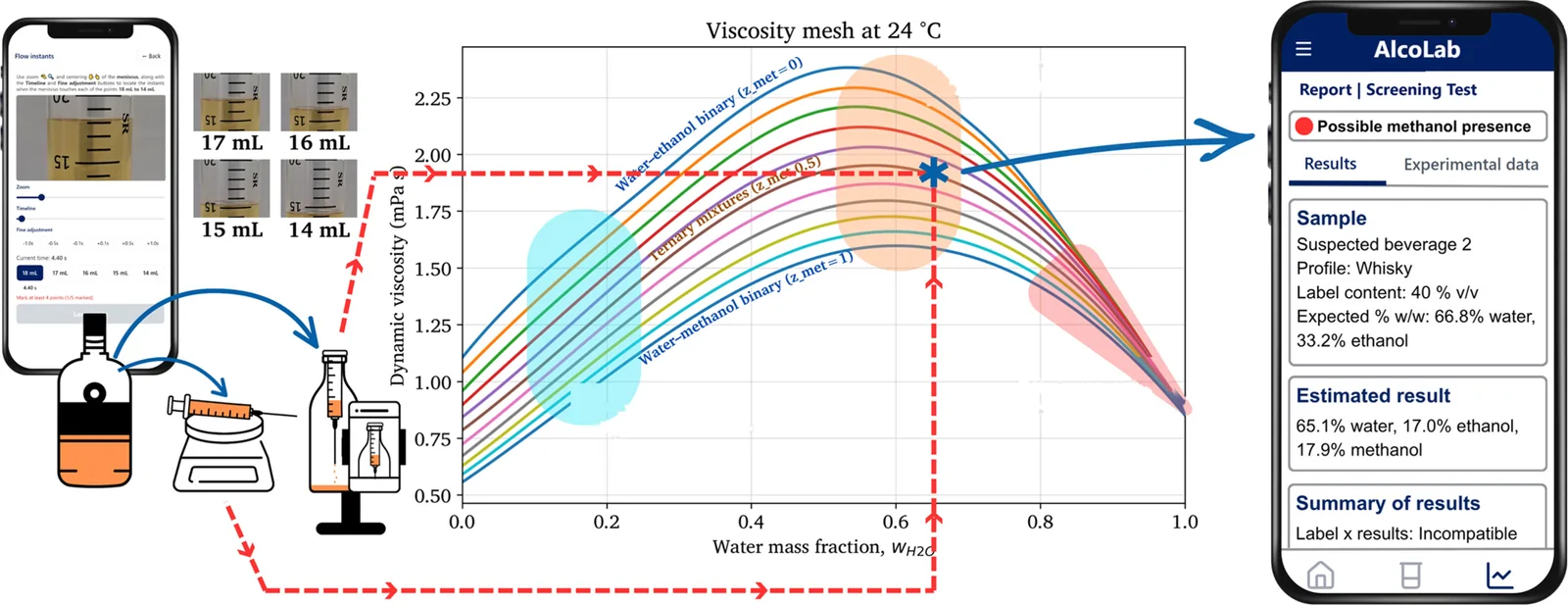

## Specifications table

**Table:** 

Subject area	Analytical Chemistry
More specific subject area	Field analytical chemistry; methanol screening in hydroalcoholic mixtures (distilled spirits and commercial ethanol solutions)
Name of your method	AlcoLab: semiquantitative methanol screening by density and syringe-flow measurements
Name and reference of original method	n/a
Resource availability	Application: alcolab.org. Source code: github.com/diegoanapolis/alcolab (GNU AGPL-3.0 license; release v1.0.0, archived at https://doi.org/10.5281/zenodo.20573733). Reproducible package of scripts and data: Zenodo, DOI 10.5281/zenodo.20574038. Materials: 20-mL disposable syringe with a 22-gauge needle; balance with 0.1 g readability (or hydrometer); smartphone or webcam.

## Background

Methanol poisoning from alcoholic beverages has regained international attention in recent outbreaks, prompting public health alerts across several countries of the Americas [1,2]. During the 2025 crisis in Brazil, investigations found that part of the methanol used to adulterate beverages had originated from diverted, adulterated fuel ethanol, showing that distinct regulatory chains (fuels and beverages) can be linked through criminal activity [3,4]. Methanol cannot be detected by consumers through color, odor, or taste, yet it is highly toxic: it causes metabolic acidosis and optic nerve injury, which can lead to permanent blindness or death [5,6]. Fatal poisoning events linked to adulterated beverages have been documented in multiple countries over several decades [[7], [8], [9]]. Fuel-ethanol adulteration with methanol is itself documented and instrumentally detectable [10,11], and methanol price fluctuations create an economic context that may favor illicit adulteration in hydroalcoholic supply chains [12].

Density is the quantity traditionally used to measure the total alcohol content of hydroalcoholic solutions. Its relationship with alcohol content in the water–ethanol system is smooth and monotonic, and it has underpinned alcoholometry for more than a century, from glass alcoholometers to benchtop density meters [13,14]. Its fundamental limitation, however, is that it cannot distinguish ethanol from methanol, whose densities are nearly identical (0.789 and 0.791 g cm^−3^, respectively) [15]. When a mixture can be assumed to consist essentially of water and ethanol, density alone is sufficient; otherwise, many water–ethanol–methanol compositions remain possible.

Responding reliably to the presence of methanol therefore requires confirmatory reference methods: gas chromatography with flame ionization detection or mass spectrometry [16,17], nuclear magnetic resonance, and Raman spectroscopy [[18], [19], [20]]. Reliable as they are, these methods are costly, depend on laboratory infrastructure and specialized personnel, and are not always available where enforcement is decentralized or screening is carried out in the field. For the end consumer, they remain effectively out of reach. Colorimetric alternatives—some already integrated with apps—lower the cost but still rely on chemical reagents, some of which are hazardous, such as concentrated sulfuric acid [[21], [22], [23], [24], [25], [26]].

A distinct and rapidly advancing line of work addresses the same problem with dedicated portable instrumentation. Piezoelectric transduction has been applied to this matrix: a quartz crystal microbalance coated with a metal–phenolic film detects methanol in alcoholic beverages under saturated vapor conditions, with suppressed cross-sensitivity to water and other interferents [27]. A handheld chemoresistive detector has undergone ISO-standardized interlaboratory validation across 17 independent participants, reaching accuracy comparable to gas chromatography [28]. Portable Raman spectroscopy has likewise been used for non-destructive determination of ethanol and toxic alcohols in spirits [29], and ion mobility spectrometry has been applied to the rapid, direct determination of methanol in alcoholic beverages [30]. These approaches achieve selectivity and detection limits that the present method does not approach, and they define the performance benchmark for the field. They do, however, require a functionalized sensor or a dedicated reader, which places them in a different cost and distribution regime.

The gap addressed here is therefore narrower than an absence of methods: few reported approaches combine reagent-free operation, near-zero consumable cost, materials already available in ordinary households, and a fully open-source implementation that can be distributed at no marginal cost to consumers, inspectors or frontline personnel.

Against this background, the present work introduces a simple, low-cost proof-of-concept method for the preliminary semiquantitative screening of methanol in hydroalcoholic mixtures, implemented in the open-source application AlcoLab. The approach combines two complementary quantities: density, obtained in relative terms on a common 0.1 g balance, and a viscosity-related parameter derived from the flow through a disposable syringe and needle. From these data, AlcoLab narrows the search region by density within a literature-anchored ternary viscosity grid and estimates the proportions of water, ethanol, and methanol. The method is proposed as a preliminary screening tool—to flag suspected samples, support reporting to authorities, and prioritize confirmatory analyses in enforcement settings—without replacing chromatography, which remains the confirmatory standard.

## Method details

The method was designed to require only a 20-mL disposable syringe fitted with a 22-gauge needle (a type widely available in pharmacies) and a common balance with 0.1 g readability. A hydrometer, where available, can replace the balance. A thermometer is not required for the calculation: the sample-to-water flow ratio is measured within the same session under the same thermal conditions, so the internal water reference compensates, to a first approximation, for the common thermal effect. When recorded, the temperature is used to check the thermal stability between the sample and the reference water and as quality-control (QC) metadata (recommended operating range 20–28°C); it is not an input to the inversion.

For the balance route, net liquid masses are obtained by subtracting the mass of the empty syringe–needle assembly, and relative density is calculated as the ratio of the net sample mass to the net water mass for equal nominal 20-mL fills under the same thermal conditions. AlcoLab maps this value to the apparent total-alcohol constraint using the embedded binary density tables.

### Applicability domain

The method is intended for simple, clear hydroalcoholic matrices: distilled spirits of the whisky, vodka and cachaça type and commercial ethanol solutions. Liqueurs and cream-based drinks, sweetened or flavored products, fermented beverages, and any sample with turbidity, suspended solids, pulp, substantial added non-volatile ingredients, or intense artificial coloration falls outside this domain and must not be analyzed: their non-volatile solutes bias the density reading, and their flow behavior is not represented by the water–ethanol–methanol grid. These pre-analytical exclusion criteria are applied by visual inspection before any measurement. Even within the domain, congeners and minor constituents of distilled beverages can perturb both measurements and may contribute to false positives; a flagged sample therefore always requires chromatographic confirmation, and a negative screening result is not proof of the absence of methanol. For these reasons, the compositional output is reported throughout as an *apparent ternary-equivalent composition*, not as an absolute determination.

Operationally, the smartphone serves as the video camera and may also host the AlcoLab PWA in its browser. The user records the paired water and sample drains and, in AlcoLab’s frame-marking interface, selects the frames at which the meniscus crosses the 18, 17, 16, 15 and 14 mL marks. The application checks the markings, fits volume against time to obtain $t_{\text{water}}$, $t_{\text{sample}}$ and $R^{2}$, calculates $A_{\text{obs}}$, combines this response with the density-derived constraint $w_{\rho }$, performs the ternary inversion and statistical interpretation, and returns the point estimate, plausible alternatives, label compatibility and traffic-light flag. All numerical processing runs locally in the browser. Thus, in the workflow evaluated here, the phone does not measure syringe flow through a sensor; the application converts user-marked video crossings and density input into the screening output. Fig. 1 sets out this division of roles and the sequence of data transformations. Panel (a) covers acquisition and the constrained inversion; panel (b) covers the statistical qualification, in which the density window, the replicate flow ratios and the point estimate feed an adaptive hypothesis set, an uncertainty assessment and a quality check on replicate count, coefficient of variation, video $R^{2}$ and water-reference agreement before the qualified result is returned. Role tags in the figure identify whether each step is performed by the user, by the user inside AlcoLab, by the browser or by the embedded model.

**Fig. 1:**
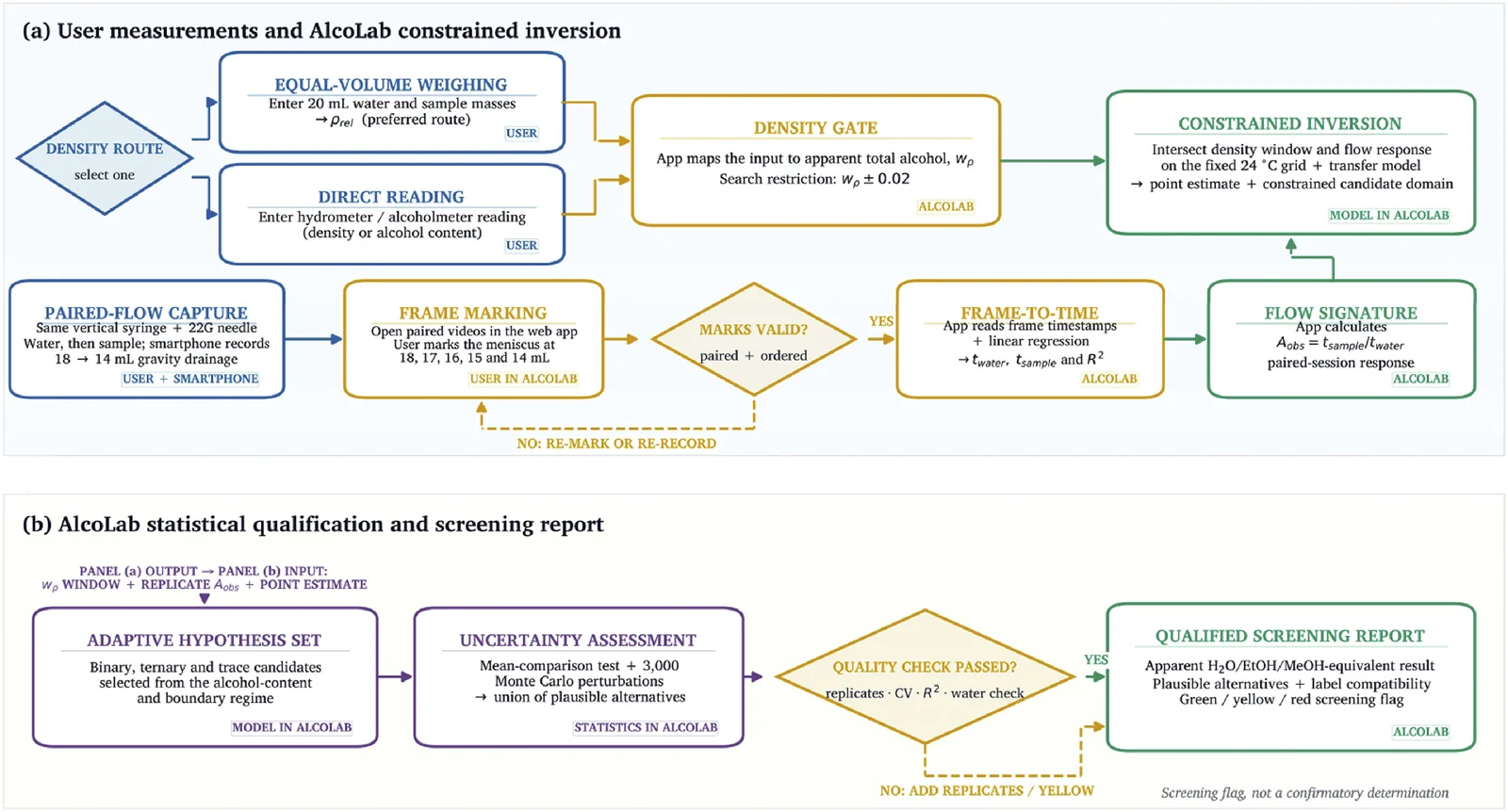
Operational chain from user measurements to the AlcoLab screening report. (a) Density route, paired video capture, frame marking and constrained inversion on the fixed 24°C grid. (b) Statistical qualification, plausible alternatives and screening flag. The smartphone records the drainage; it does not measure flow through a phone sensor.

The experimental procedure can be carried out entirely through the AlcoLab application, available at alcolab.org. Its screens, shown below, guide the user through the workflow with clear, concise instructions, while still allowing access to the full protocol at each step. Representative AlcoLab screens documenting the user-facing sequence of sample selection, data entry, measurement and result display are shown in Fig. 2; the corresponding division of roles and data transformations is defined in Fig. 1.

**Fig. 2:**
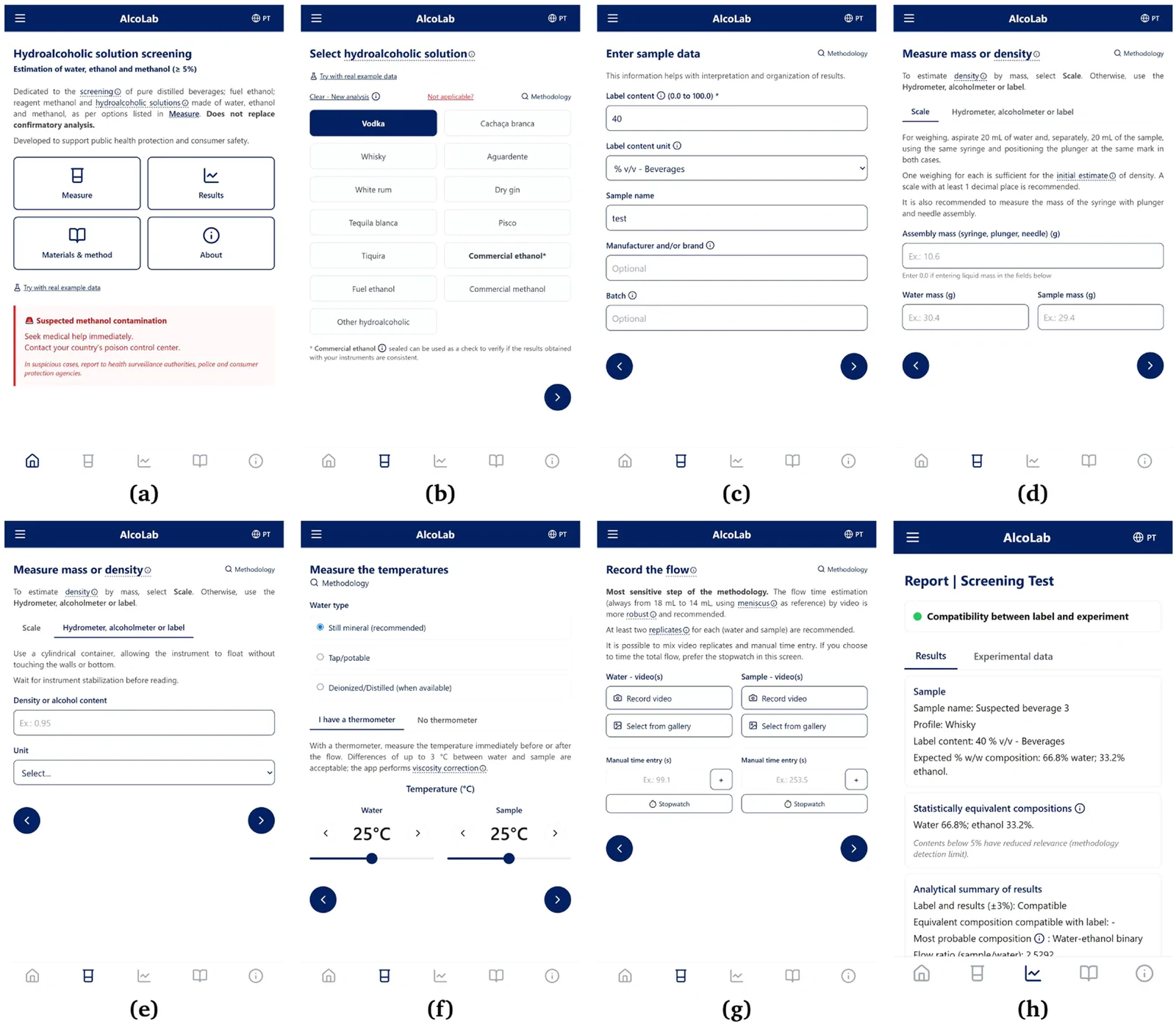
Representative AlcoLab application screens documenting the user-facing sequence from sample selection and data entry to measurement and result display (alcolab.org).

For each flow video, the user records, in a dedicated AlcoLab window, the instants at which the liquid meniscus crosses the 18, 17, 16, 15, and 14 mL marks, as illustrated in Fig. 3. Marks above 18 mL may not stay visible when the syringe rests on improvised stands such as PET bottles, and below 14 mL, the window screening described in the supplementary material showed an increase in the coefficient of variation of the flow. The 18–14 mL window was therefore adopted as an operational compromise between visibility, run duration, and reproducibility.

**Fig. 3:**
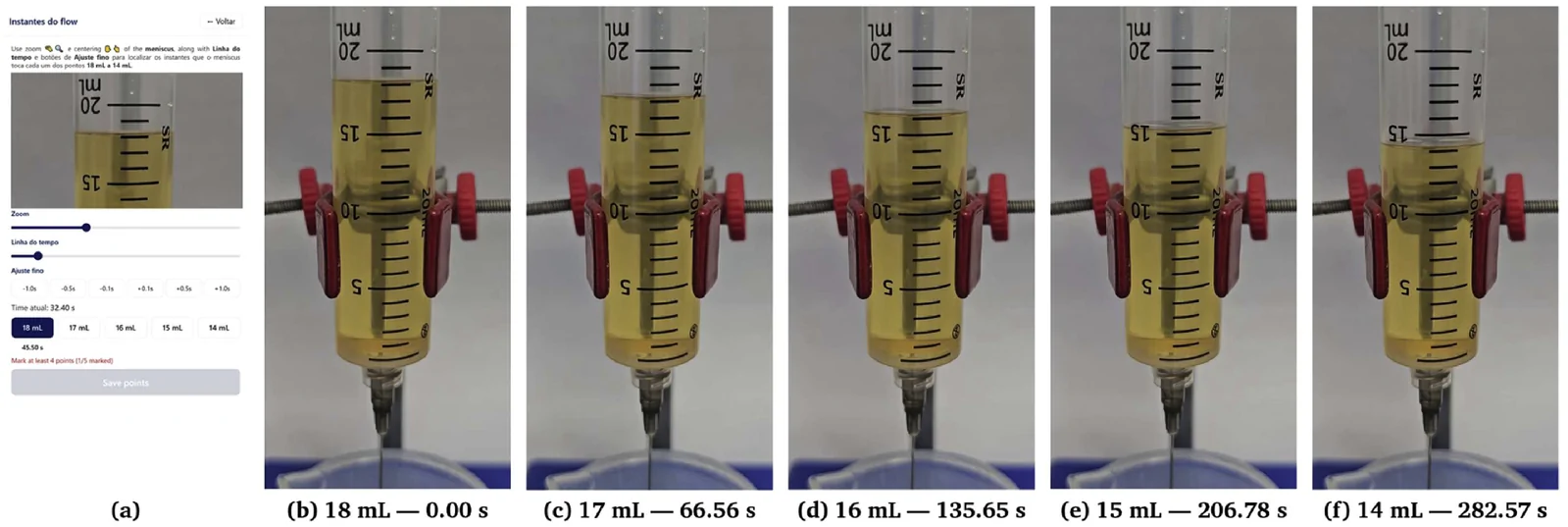
Panel (a) shows the app window for frame marking; panels (b–f) show the frames defined by operator A at which the meniscus reaches the 18, 17, 16, 15, and 14 mL marks, with the respective relative flow times. Sample: commercial whisky, 40% v/v ethanol, without methanol addition (video AlcoLab_V004 from the supplementary material; hardware configuration SR–SR; 23°C).

Some imprecision in identifying and interpreting the meniscus is acknowledged, but it is effectively mitigated by the use of relative metrics and of linear regression to obtain the times. This source of error is further minimized when the same person applies a consistent marking criterion to all frames of a given flow and to the flow video recorded for the reference water.

### Ternary grid anchored in the literature

Throughout this work, mole fractions are denoted by $x_{i}$ and mass fractions by $w_{i}$, where i is water, ethanol, or methanol. The viscosity grid is built in mole fraction, whereas the experimentally prepared compositions and the results reported to the user are expressed in mass fraction. In the inversion step, the total alcohol mass fraction is defined as $W_{A}=w_{\text{EtOH}}+w_{\text{MeOH}}$ and the methanol mass fraction in the alcohol as $z=w_{\text{MeOH}}/W_{A}$. The operational density-based estimate of this apparent total alcohol content is denoted by $w_{\rho }$.

Measuring a complete ternary grid across several compositions and temperatures, as a reference basis for the method, would require instrumentation, larger amounts of reagents, and strict control of the ambient temperature—resources unavailable to the authors in this independent study. For this reason, and given the good availability of water–alcohol binary-mixture data, the grid was instead built and assessed using data consolidated from the literature.

The data used to construct and validate the grid were obtained from six literature sources with distinct, complementary roles: constructing the binary edges, supporting low-temperature behavior, anchoring the temperature dependence of water, anchoring the ternary interior, and serving as the model-selection benchmark for the ternary interior (Table 1).

**Table 1:** Literature sources used in the construction, anchoring, model selection, and assessment of the water–ethanol–methanol viscosity grid.

Reference	System	Temperatures	Property / unit	Data type	Role in this study
González et al. [31]	water–ethanol; water–methanol	293.15; 298.15; 303.15 K	dynamic viscosity (mPa·s)	experimental table	support for the binary edges in the 20–30°C range
Khattab et al. [32]	water–ethanol	293–323 K (5 K steps)	viscosity and density (mPa·s)	experimental table	water–ethanol edge 20–50°C, especially 35–50°C
Mikhail and Kimel [33]	water–methanol	25; 30; 35; 40; 50°C	viscosity (millipoise)	experimental table	water–methanol edge 25–50°C (1 millipoise = 0.1 mPa·s)
Kestin et al. [34]	pure water	wide range	water viscosity (mPa·s)	reference table	anchoring of $\mu_{\text{water}}\left(T\right)$
Song and Peng [35]	water–methanol–ethanol (ternary)	20 °C	viscosity (mPa·s)	table/graph, digitized points	anchoring of the ternary interior
Dizechi and Marschall [36]	water–methanol–ethanol (ternary)	10; 20; 30; 40; 50°C	kinematic viscosity + density → dynamic	experimental table	model-selection benchmark for the ternary interior at 20–30°C

Starting from the literature binary edges, the grid was constructed over the 20–50°C range through the following steps.

**Step 1 — Consolidation of the binary data.** The available binary data were harmonized onto a single composition grid, converting between mass fraction and mole fraction where necessary. The computational grid was built in mole fraction from 0 to 100%, and the compositions were also kept in mass fraction for experimental preparation, validation, and reporting. The temperature grid spanned 20 to 50°C in 1°C steps, with all viscosities expressed in mPa·s.
**Step 2 — Thermal regularization of the edges.** Between the experimental anchors — 20, 25, 30, 35, 40, 45, and 50°C (water–ethanol [32,31]; water–methanol [33,31]) — the intermediate layers were generated by log-scale interpolation. The 20°C layer of the water–methanol edge, which is not available as a complete experimental layer in [33], was completed using low-temperature data from [31] together with this regularization.
**Step 3 — Compositional reconstruction of the edges (PCHIP).** At each integer temperature, every edge was reconstructed by shape-preserving monotone PCHIP interpolation (*piecewise cubic Hermite interpolating polynomial*) applied to lnμ as a function of the water mole fraction $x_{W}$, with endpoints anchored at $x_{W}=0$ and $x_{W}=1$. This yields two smooth viscosity functions, $\mu_{H_{2}O--\text{EtOH}}\left(x_{W},T\right)$ and $\mu_{H_{2}O--\text{MeOH}}\left(x_{W},T\right)$, which serve as the edges of the ternary grid. The reconstruction preserves the contrast between pure ethanol and methanol at 20°C (≈1.2 and 0.55 mPa·s, versus ≈1.0 mPa·s for water) as well as the positive viscosity deviation of the aqueous mixtures, with pronounced maxima at intermediate composition—a phenomenon generally attributed in the literature to the reorganization of the hydrogen-bond network in water–alcohol mixtures [32,31].
**Step 4 — Sampling of the ternary interior.** The interior of the triangle was sampled on a regular simplex with a 1 mol% step ($x_{W}\%+x_{\text{MeOH}}\%+x_{\text{EtOH}}\%=100$). At each node, the water mole fraction $x_{W}$ and the methanol mole fraction within the set of alcohols are defined as:
  $$z_{x}=\frac{x_{\text{MeOH}}}{x_{\text{EtOH}}+x_{\text{MeOH}}}$$

where $z_{x}=0$ recovers the water–ethanol edge and $z_{x}=1$ the water–methanol edge. In the inversion and reporting step, the candidate compositions are converted to mass fractions and expressed through $W_{A}$ and z.

**Step 5 — Ternary viscosity (log-geometric mixing + excess).** The viscosity at each interior node is obtained from a log-geometric mixing of the edges, plus an excess term:
  (1) $$\text{ln}\mu_{\text{tern}}\left(x_{W},T\right)=\left(1-z_{x}\right)\;\text{ln}\mu_{\text{Et}}\left(x_{W},T\right)+z_{x}\;\text{ln}\mu_{\text{Me}}\left(x_{W},T\right)+z_{x}\left(1-z_{x}\right)\;\Delta \left(x_{W},T\right)$$

Again, $z_{x}=0$ recovers the water–ethanol edge and $z_{x}=1$ the water–methanol edge. With Δ=0, Eq. (1) reduces to the ideal log-linear limit, analogous to the Arrhenius viscosity mixing rule. In its complete form, Eq. (1) can be interpreted as a Grunberg–Nissan-type expression, in which the term $z_{x}\left(1-z_{x}\right)\;\Delta \left(x_{W},T\right)$ acts as the interaction/excess term in the ternary interior [37].

**Step 6 — Form of the excess term.** The factor $z_{x}\left(1-z_{x}\right)$ cancels the excess term exactly at the binary edges ($z_{x}=0$ and $z_{x}=1$), so that it operates only in the ternary interior — the structure of a first-order truncated Redlich–Kister-type correction [38]. Two distinct realizations of $\Delta (x_{W},T)$ are used in this work and must be distinguished. The *reference computational grid*, from which the binary edges are inherited, employs the canonical parameterization with λ=0.20 (intensity) and α=0.30 (magnitude bound), with PCHIP smoothing of the correction field as a function of $x_{W}$ at each temperature. The *deployed grid*, used by the application and in all results reported here, retains those binary edges but replaces the ternary interior by an excess term fitted to literature data, as described in Step 8.
**Step 7 — Regularizing bound of the ternary correction.** A bound proportional to the contrast between the two binary edges is available to prevent the correction from introducing artificial curvature:
  (2) $$\left|\Delta \left(x_{W},T\right)\left|\leq \alpha \;\right|\text{ln}\mu_{\text{Et}}\left(x_{W},T\right)-\text{ln}\mu_{\text{Me}}\left(x_{W},T\right)\right|$$

In the canonical parameterization this bound is active with α=0.30. In the deployed grid it is *not* active: the fitted correction of Step 8 remains, by itself, within the physical bound over the evaluation set, so no clipping is applied (see Step 8).

**Step 8 — Anchoring and selection of the ternary interior.** The ternary interior was anchored at ten points from [35] at 20°C, digitized from Figure 7 of that article. Twenty-three candidate formulations of $\Delta (x_{W},T)$ — the canonical Redlich–Kister term, the log-geometric limit (Δ=0), a rescaled canonical term, and twenty ridge-regularized low-order polynomials in $\left(x_{W},T\right)$ — were fitted to those ten points, and the final formulation was selected by minimizing the mean absolute percentage error (MAPE) over the 46 ternary points of [36] at 20 and 30°C. Because the same dataset selected the candidate and reports the resulting error, that error is a *model-selection benchmark* rather than an untouched external validation; the associated optimism is quantified in *Method validation*. The winning formulation, its verification against the distributed grid file (agreement to $6\times 10^{-8}$) and the inactivity of the bound of Step 7 are summarized in Table 2; the full candidate ranking and the original grid-construction scripts are in the deposited supplementary package; the nested-resampling check added during the revision is described in *Method validation*.

**Table 2:** Structure and parameters of the water–ethanol–methanol viscosity grid. Binary edges are inherited from the canonical reference grid; the ternary interior is the fitted formulation selected in Step 8.

Parameter	Value
Temperature range (computational grid)	20–50°C
Quantitative assessment range (operational)	20–30°C
Temperature step	1°C
Composition basis	mole fraction
Composition step	1 mol%
Composition nodes per temperature	5151
Temperature layers (20–50°C)	31
Approximate total of nodes	≈ 159,681
Binary-edge interpolation	PCHIP in lnμ vs. $x_{W}$
Excess-term coefficients of the canonical grid (interior; not used in the deployed interior)	λ=0.20; α=0.30
Ternary-interior excess term (deployed grid)	ridge-regularized polynomial in $\left(x_{W},T\right)$
Ridge regularization parameter	10.0
Magnitude bound α in the deployed grid	not active (see Steps 7 and 8)
Viscosity range at 20°C	≈ 0.585 to 2.705 mPa·s
Fitting of the ternary interior	Song and Peng [35] (10 points, 20°C)
Model selection among 23 candidates	Dizechi and Marschall [36] (46 points, 20 and 30°C)
Selection-benchmark error	MAE ≈ 0.043 mPa·s; MAPE ≈ 2.47% (n = 46)
Nested-CV estimate of the same procedure	MAPE ≈ 2.47%; selection optimism ≈ 0.00 p.p.
Independent check (molecular simulation)	Parez et al. [39] (7 ternary points, 298 K)
Independent-check error	MAE ≈ 0.096 mPa·s; MAPE ≈ 6.80% (n = 7)

Fig. 4 shows the fixed 24°C layer actually queried by the deployed inversion and marks the ten interior compositions of [35] that anchored the ternary correction. The grid was constructed in mole fraction but is presented in coordinates converted to water mass fraction and methanol mass fraction in the alcohol (z), to facilitate interpretation. The circles identify compositional support only: those viscosities were measured at 20°C and are not direct observations of the 24°C layer.

**Fig. 4:**
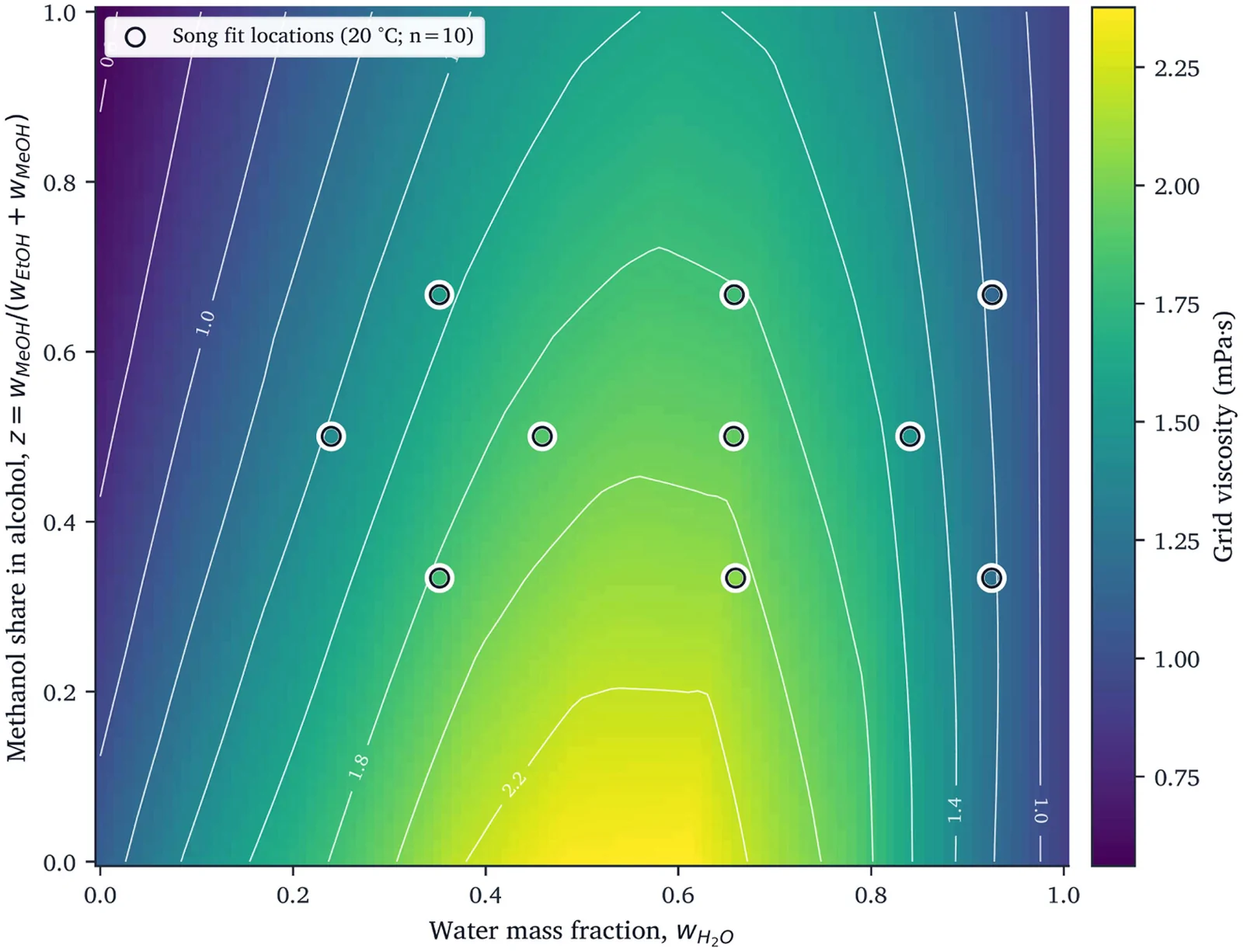
Fixed 24°C viscosity grid used by the deployed inversion. Color and white contours at 0.2 mPa·s intervals give grid viscosity as a function of water mass fraction and the methanol share within total alcohol, z. Open circles mark the compositional locations of the ten [35] anchor points. Grid geometry only; color separation is not an experimentally validated detection limit.

### Empirical transfer model between literature viscosity and syringe-flow measurements

The grid from the previous section provides the literature reference viscosity, whereas the syringe–needle experiment provides a measured flow time. This section defines the forward model that links the two: given a candidate composition—total alcohol mass fraction $W_{A}$ and methanol mass fraction in the alcohol z—it predicts the flow-time ratio that the syringe–needle–smartphone assembly would produce. Both quantities enter as dimensionless ratios to a water reference—the measured flow time to the flow time of the paired water run, and the grid viscosity to the grid viscosity of water on the same fixed 24°C layer—as described below.

The model was calibrated with hydroalcoholic mixtures of known composition, prepared gravimetrically from analytical-grade ethanol, analytical-grade methanol, and ultrapure (Milli-Q) water on a calibrated analytical balance. These compositions spanned pure liquids, water–ethanol and water–methanol binaries, and water–ethanol–methanol ternaries in the range of interest for spirits, as well as commercial ethanol and distilled spirits. All replicates were processed by regression over the 18–14 mL window selected in the screening described above. Table 3 summarizes the 54 eligible replicates, across 19 compositions, used in the fit; the per-replicate data are provided in the supplementary material.

**Table 3:** Calibration hydroalcoholic mixtures eligible in the 18–14 mL window (regression), with individual component mass fractions, number of replicates, and median flow time. Total: 54 replicates across 19 compositions.

$w_{W}$	$w_{\text{EtOH}}$	$w_{\text{MeOH}}$	n	Median T (°C)	Median flow time (s)
0.000	0.000	1.000	4	20	86.5
0.000	1.000	0.000	4	20	174.0
0.054	0.946	0.000	2	20	201.5
0.133	0.867	0.000	2	20	231.5
0.201	0.000	0.799	2	20	142.7
0.237	0.763	0.000	2	20	275.3
0.414	0.345	0.241	1	20	252.0
0.418	0.357	0.225	3	24	245.6
0.419	0.000	0.581	2	20	193.5
0.476	0.524	0.000	2	20	327.8
0.539	0.258	0.203	5	21	262.0
0.539	0.461	0.000	5	23	347.4
0.594	0.000	0.406	2	20	211.6
0.668	0.166	0.166	3	23	257.6
0.668	0.332	0.000	4	23	299.1
0.677	0.323	0.000	4	21	290.0
0.679	0.155	0.166	3	23	258.3
0.690	0.310	0.000	3	23	288.3
0.808	0.000	0.192	1	20	195.3

#### Real commercial matrices in the calibration set

Because the composition of the eligible replicates is reported in Table 3 only as ternary mass fractions, the proportion measured in real products is not apparent from that table and is stated here. Of the 54 eligible replicates, **30 (56%) were replicate measurements performed in commercial products**—measurements, not distinct products: 22 in distilled spirits — whisky (7), vodka (6) and cachaça (9) — and 8 in commercial ethanol at 54 °GL. Within the 22 spirit replicates, 11 were measured as purchased and 11 after methanol was added gravimetrically; of the 8 commercial-ethanol replicates, 5 were unspiked and 3 spiked. The spiked commercial matrices carry the lowest methanol fractions of the whole eligible set (mass fractions of 0.166, 0.203 and 0.225), so the real-matrix measurements are concentrated at the lower end of the evaluated positive range rather than at the idealized extremes. The matrix identity and the spiked/unspiked condition of each replicate are given in the supplementary material.

These measurements are best read as *proof-of-concept evidence that the method responds in real matrices*, not as external validation: the products were part of the development set, they comprise three spirit categories from single purchases rather than a sampled cohort, and no paired chromatographic determination was performed. Accordingly, and because non-volatile solutes can bias a direct density reading of an undistilled beverage, the compositional output is reported throughout as an *apparent ternary-equivalent composition* rather than as an absolute compositional determination.

Within those bounds, conditional prediction errors were not larger in the commercial matrices than in the idealized preparations. Across the blocked evaluation schemes described in Method validation, the mean absolute error of the methanol mass fraction was 0.026 in the commercial-spirit replicates and 0.015 in the commercial-ethanol replicates, against 0.029 in the prepared mixtures and reagents; the descriptive false-positive fraction among scheme-specific predictions from methanol-free replicates was likewise not higher in the commercial matrices.

Fig. 5 summarizes the provenance and methanol status of the 54 eligible video-replicate measurements, whereas Table 3 reports their ternary compositions and replicate counts. Counts in the figure represent replicate measurements rather than distinct commercial products.

**Fig. 5:**
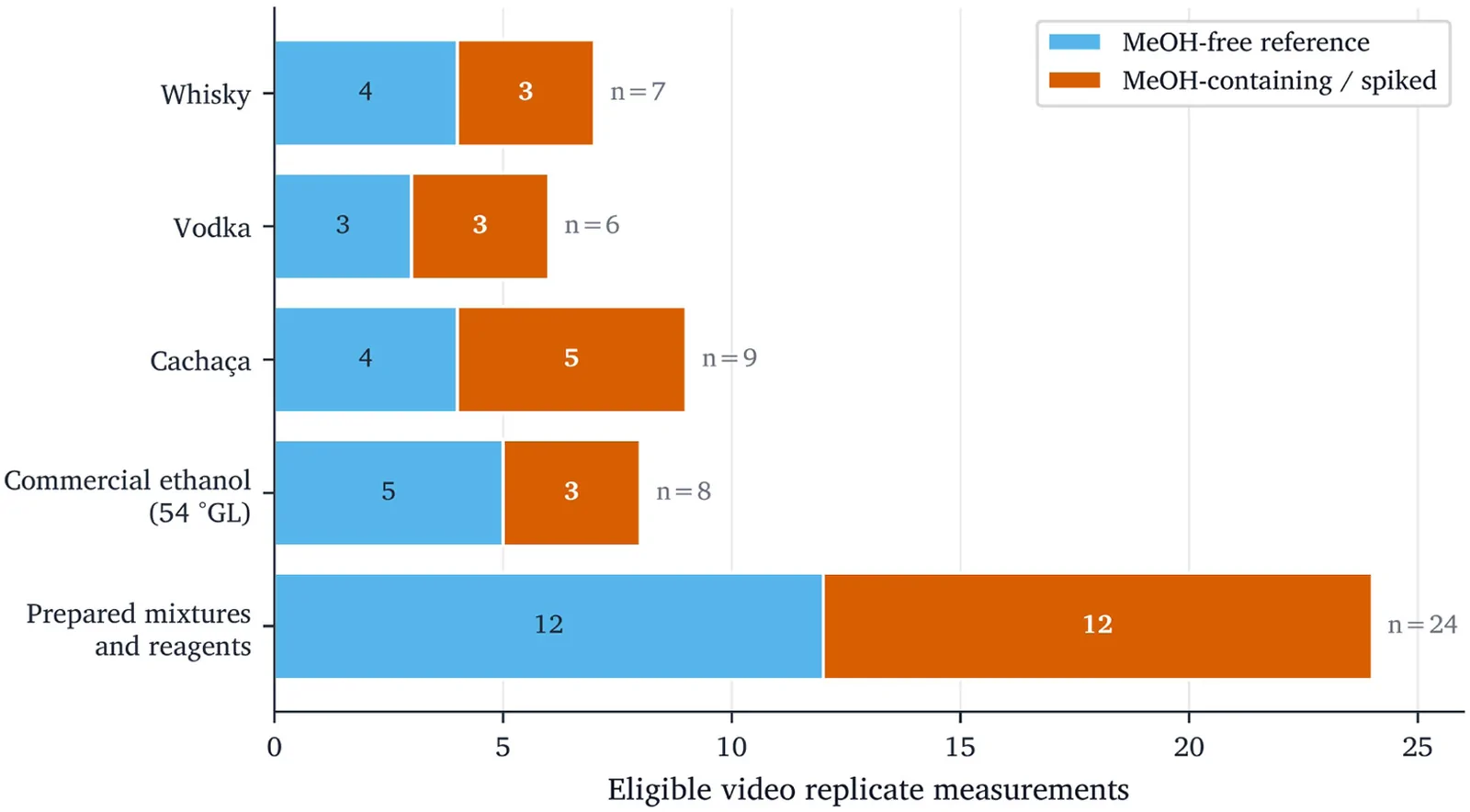
Matrices represented in the 54-replicate eligible video dataset. Counts refer to video-replicate measurements, not distinct products. Twenty-two measurements were performed in whisky, vodka and cachaça categories and eight in commercial ethanol (54 °GL); the remaining 24 were prepared mixtures or reagents. The commercial measurements belonged to model development and had no paired chromatographic reference.

The model starts from the physics of capillary flow. The observed flow-time ratio is the ratio of the flow times of the sample and of the water, measured in the same session on the same syringe–needle–operator assembly; the water measurement performed in the same session already corrects this quantity for variations in the assembly and day-to-day experimental conditions:

(3) $$A_{\text{obs}}=\frac{t_{\text{sample}}}{t_{\text{water},\text{local}}}$$

Because $A_{\text{obs}}$ is a sample-to-water ratio measured within the same session and at the same temperature, the thermal effect common to the experiment—and the assembly geometry—cancels, to first order, in the quotient itself, so no explicit thermal correction is applied to the observed ratio. A formulation that normalized the ratio to 20°C was evaluated in an exploratory stage, did not improve the results, and was not retained (see Method validation). The grid is queried at a fixed layer of 24°C. The *range* of candidate layers (20–24°C) was set a priori by the working temperature of the calibration sessions, but the *choice* of 24°C within that range was made post hoc, by comparing layers on the same blocked-evaluation set. It is therefore reported throughout as an exploratory selection rather than as a pre-specified parameter (see Method validation).

In gravity-driven capillary flow, the flow time depends on both viscosity and density, because the driving pressure is hydrostatic. To a first approximation, and for a fixed geometry, the temporal response approaches a kinematic quantity proportional to μ/ρ rather than a direct measure of dynamic viscosity; the simple ratio of times should therefore not be read as a relative dynamic viscosity. Here, $A_{\text{obs}}$ is treated as an operational flow-time ratio, calibrated against the dynamic-viscosity ratio of the reference grid:

(4) $$\mu_{\text{ratio},24}\left(W_{A},z\right)=\frac{\mu_{\text{lit}}\left(W_{A},z,24\right)}{\mu_{\text{water}}\left(24\right)}$$

where $\mu_{\text{lit}}$ is taken from the Song2008-anchored literature grid and $\mu_{\text{water}}$ from the viscosity of water [34]. In practice, the syringe–needle assembly departs systematically from an ideal capillary—through the effect of density on the hydrostatic pressure, the change in the liquid column during flow, and entrance and exit effects in the needle—non-idealities well recognized in simple capillary viscometry [40]. Rather than interpreting the ratio of times as an absolute viscosity, AlcoLab retains the grid as a physicochemical reference and fits an empirical transfer function between the operational flow response and the reference dynamic-viscosity ratio:

(5) $$A_{\text{pred},24}\left(W_{A},z\right)=\left(1+B\;W_{A}+C\;W_{A}^{2}\right)\;\mu_{\text{ratio},24}\left(W_{A},z\right)$$

B and C are the model’s two compositional parameters, fitted to the experimental data; together they define an empirical transfer function between the reference dynamic-viscosity ratio and the operational response of the syringe–needle–smartphone assembly, for the volume window and processing used in this study. The low-order form was chosen for parsimony and interpretability: the empirical factor equals 1 in water ($W_{A}=0$, so $A_{\text{pred},24}=\mu_{\text{ratio},24}$) and varies smoothly with the total alcohol mass fraction. The term mainly absorbs the conversion between a kinematic-type temporal response and the dynamic-viscosity ratio of the grid, together with residuals associated with the assembly geometry and its entrance and exit losses.

Because the observable is a same-session ratio and the grid is queried at a fixed layer, temperature is not an input to the inversion. When recorded, it is kept as quality-control metadata: the session water-flow response can be compared with the water response expected for the recorded temperature, both checking the thermal stability between sample and reference water and flagging sessions outside the recommended operating range (20–28°C). This QC diagnostic—including the empirical relationship between the water flow time and temperature, $t_{\text{water}}\approx k_{\text{HW}}\left(T\right)\;\mu_{\text{water}}\left(T\right)/k_{\text{ideal}}$, with $k_{\text{HW}}\left(T\right)=-0.002845\;T+0.6684$ and $k_{\text{ideal}}=0.0050627$ for the assembly used—is documented in the supplementary material and does not affect the inversion result.

The protocol always requires recording a water flow in the same session as the sample, since this is what anchors Eqs. (3) and (4). In this prototype, B and C were treated as global parameters for the 20-mL syringe / 22-gauge needle configuration and the 18–14 mL window used here; their universality across other manufacturers, batches, geometries and processing schemes remains to be demonstrated, and other hardware configurations will likely require recalibration. An extended form with a multiplicative day-specific water correction (Vsin) was evaluated in an exploratory stage and not retained, as the session correction already enters $A_{\text{obs}}$; its parameters are given in the supplementary material.

B and C were obtained by minimizing the residual between $A_{\text{pred},24}$ and $A_{\text{obs}}$ on a logarithmic scale, with mild regularization, over the 54 eligible replicates of the 18–14 mL window (Table 3); the fitted values are B=+0.082591 and C=+0.134034.

The core transfer model and the compositional grid-search inversion—Eqs. (3)–(6)—are implemented in the deposited script final_model.py.

### Ternary inversion and the application’s calculation workflow

The previous sections solve the forward problem, mapping a known composition to its predicted flow-time ratio. The application solves the inverse problem: from the user’s relative-density and paired water/sample flow measurements, with temperature retained only as quality-control metadata, it produces the screening outputs, namely an estimate of the apparent total alcohol content, the methanol fraction in the alcohol, and a traffic-light result. Fig. 6 illustrates why the flow response alone is non-unique and how the density-derived total-alcohol restriction separates calculated compositions with similar fixed-grid viscosity but markedly different total-alcohol contents.

**Fig. 6:**
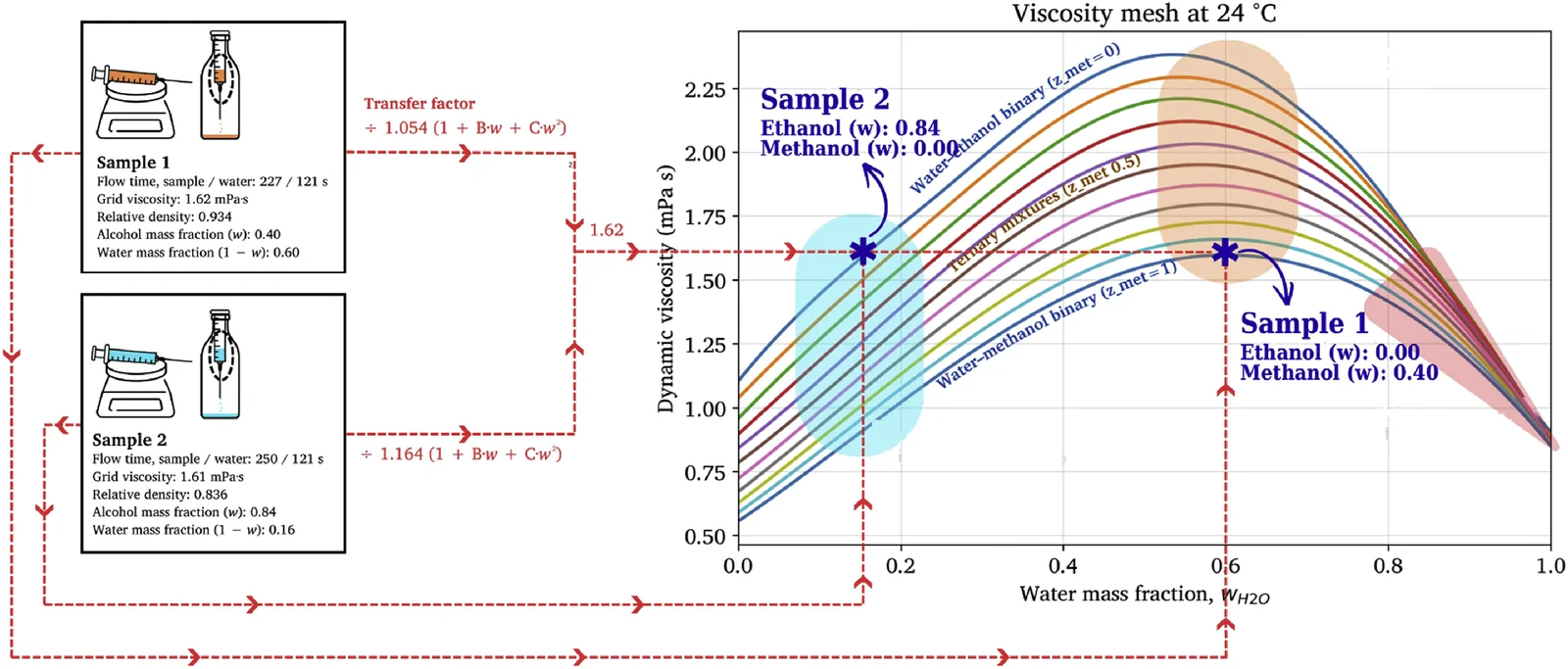
Density resolves non-unique flow responses in two calculated illustrative compositions. The water–methanol and water–ethanol examples reach nearly the same fixed-grid viscosity (≈ 1.62 mPa·s) after the transfer correction despite different flow-time ratios, but their relative densities (≈ 0.934 and ≈ 0.836) place them in distinct total-alcohol windows. Both marked points are calculated illustrations, not experimental samples.

In Fig. 6, each grid curve on the fixed 24°C layer fixes a methanol mass fraction in the alcohol z, from the water–ethanol binary (z=0) to the water–methanol binary (z=1). The figure illustrates the central difficulty of the inversion: the two calculated compositions marked with an asterisk reach the same corrected response on the grid scale (≈ 1.62 mPa·s, dashed line) despite having different flow-time ratios. This happens because the model’s empirical transfer function, the factor $1+B\;W_{A}+C\;W_{A}^{2}$, depends on the total alcohol mass fraction, so different times at different alcohol contents can map to the same corrected response. Moreover, a given viscosity on the grid corresponds not to a single point but to a contour line of many compatible compositions. The two compositions shown represent two such possibilities, and they differ markedly: the water–methanol example ($w_{H_{2}O}=0.60$, $w_{\text{MeOH}}=0.40$) lies in the total-alcohol range of distilled spirits but contains only methanol as its alcohol, whereas the water–ethanol example ($w_{H_{2}O}=0.16$, $w_{\text{EtOH}}=0.84$) resembles a concentrated ethanol solution slightly diluted with water. Both are calculated illustrations, not experimentally measured samples.

Viscosity alone cannot resolve this ambiguity, and density alone does not uniquely determine the ternary composition either. The solution proposed here lies in combining the two quantities. The relative density, obtained by weighing a calibrated volume, gives an operational estimate of the apparent total alcohol content—and the two compositions have clearly distinct relative densities (approximately 0.934 for the water–methanol example and 0.836 for the water–ethanol example in Fig. 6). In the application, this estimate acts as a region filter: rather than fixing a single composition, it restricts the search to a narrow window of the grid (±2 percentage points) around $w_{\rho }$. Within that window, the operational flow response then selects the most compatible ethanol-to-methanol proportion.

Formally, within the window defined by the operational density-based estimate $w_{\rho }$, the application seeks the composition $\left(W_{A},z\right)$ whose predicted flow-time ratio is closest to the observed one:

(6) $$\left({\hat{W}}_{A},\hat{z}\right)=\text{arg}\underset{\begin{array}{c} W_{A}\in \left[w_{\rho }-\Delta W_{A},\Delta w_{\rho }+\Delta W_{A}\right]\\ z\in \left[0,\;1\right] \end{array}}{\text{min}}\left|A_{\text{pred},24}\left(W_{A},z\right)-A_{\text{obs}}\right|$$

The search sweeps the methanol mass fraction in the alcohol z over the whole interval [0,1] and, for each z, optimizes the total alcohol mass fraction $W_{A}$ within the window; the pair $\left({\hat{W}}_{A},\hat{z}\right)$ with the smallest deviation is the point estimate.

This combination resolves an ambiguity that neither quantity could resolve on its own: density constrains how much apparent total alcohol is compatible with the sample, and the operational flow response, within that range, indicates which ethanol-to-methanol proportion best explains the observed response. The point composition $\left({\hat{W}}_{A},\hat{z}\right)$ is the starting point for the statistical layer and the traffic-light logic described below.

The highlighted regions in the figure illustrate the geometric separation between grid curves; they are not experimentally validated detection limits, nor performance estimates for those matrices.

The function invert_final_model in the supplementary script final_model.py implements the sweep over z, the optimization of $W_{A}$ within the window defined by $w_{\rho }$, and the selection of the composition that minimizes the deviation between $A_{\text{pred},24}$ and $A_{\text{obs}}$.

### Statistical pre-interpretation layer and traffic-light logic

The inversion of Eq. (6) returns the point composition most compatible with the measurements, but this alone is not enough to support responsible use. Simple, low-cost measurements—weighing, video recording, meniscus marking, and the variability between replicates—carry uncertainty, and different yet nearby compositions can yield statistically indistinguishable flow-time ratios. The statistical layer has no confirmatory role; it provides an accessible, interpretable preview of the result, so that the raw estimate does not hide from the user other viscometrically plausible compositions, especially where binaries and ternaries give similar responses.

For each sample, the layer compares four hypothesis families, all evaluated within the alcohol-content range constrained by density: bin_etoh, the water–ethanol binary (z=0); bin_meoh, the water–methanol binary (z=1); ternary, with z free; and trace, compositions containing a small fraction (≈1–6%) of one alcohol in the other, intended to capture low-level adulteration. For each family, the layer identifies the composition that best explains the observed flow-time ratio. Three criteria are then combined.

The test of the mean compares the observed mean flow-time ratio with the predicted one for each hypothesis using a two-sided test—a Student’s t-test when two or more replicates are available, or a normal approximation with a reference standard deviation when only one is—at the conservative level α=0.01. Hypotheses that are not rejected are treated as statistically compatible with the observation, within the adopted experimental uncertainty. Because the layer is exploratory and conservative, these tests are not used to declare the presence or absence of methanol in a confirmatory way, but to assemble a set of compatible hypotheses for the user.

The Monte Carlo simulation propagates the operational uncertainty of the flow-time ratio [41,42]: N=3000 values are drawn around the observed mean, assuming a normal distribution with a standard deviation estimated from the replicates; when only one replicate is available, the reference standard deviation defined in the supplementary material is used. For each simulated value the most compatible hypothesis is recorded, so that the reported value for a hypothesis is the fraction of simulations in which it is selected under the assumed noise model. These values are *selection frequencies under perturbation*, not posterior probabilities that a hypothesis is true; they rank and communicate compatible alternatives, not belief. The generator’s seed is derived from the sample identifier, ensuring reproducibility. In this version the simulation propagates the uncertainty of the flow-time ratio, while the uncertainties of density, temperature, the literature grid, and the empirical model are handled in a simplified way or incorporated indirectly through the search window and the conditional blocked evaluation.

The union rule then defines the final set of compatible hypotheses as the union of those not rejected by the test of the mean, those with a Monte Carlo selection frequency above a threshold, and the best point hypothesis—ensuring that the best estimate and the plausible alternatives are always shown rather than masked.

The value N=3000 comes from a convergence study of the simulation, reported in the supplementary material: against an N=200,000 reference, the maximum mean selection-frequency error falls to ≈0.005 and the 95th percentile to ≈0.011, with high stability of the most probable class; increasing N further yields only a small numerical gain relative to the processing cost in the browser.

The plausible set—the hypotheses retained by this rule—is always reported as a conservative safeguard against two symmetric errors. A result compatible with the water–ethanol binary curve may be indistinguishable from a mixture with a small methanol fraction (≈3%), and both compositions are then presented as possible. Conversely, a point estimate with low methanol may be indistinguishable from the nearest water–ethanol binary composition on the grid; reporting the full set of compatible hypotheses then avoids incorrectly flagging a beverage whose low “methanol” indication is merely a measurement fluctuation around the binary edge.

The ambiguity that would genuinely require caution—alcohol content tuned precisely to the label and methanol at a very low fraction—cannot be ruled out and is treated as a limitation of the method. In the documented outbreaks, however, the concerning samples showed large compositional deviations, with high methanol fractions and/or no ethanol—the scenario in which the combination of density and flow tends to be most discriminating—and either condition alone (label incompatibility, or statistically dominant methanol) is enough for the application to issue an alert.

Table 4 illustrates the layer’s output for a methanol-containing sample and for a water–ethanol beverage without methanol, showing the prudential behavior described above.

**Table 4:** Examples of statistical-layer output (Monte Carlo selection frequencies, *N* = 3000) for a sample containing methanol and a water-ethanol sample without methanol.

Scenario	Hypothesis	Selection frequency
Sample containing methanol	ternary	1.000
	water-ethanol binary	0.000
	water-methanol binary	0.000
Water-ethanol beverage without methanol	water-ethanol binary	0.948
	ternary (trace)	0.053
	water-methanol binary	0.000

The result is communicated through a traffic-light classification logic—green, yellow, red—that integrates three components:

• *experimental quality* — number of replicates, coefficient of variation, regression fit, and agreement between the water-flow time and its reference value;
• *analytical compatibility with the label* — compatible, incompatible, or inconclusive, from the statistical layer;
• *semantics by sample type* — the result is interpreted according to whether the sample is a beverage or a commercial ethanol solution.

The logic is conservative by construction: red is triggered only when the experimental evidence is of good quality, the statistical decision is robust, and the context allows a firm conclusion—for example, methanol in a distilled spirit—while borderline cases fall into yellow, with a recommendation to repeat the assays or add replicates. Fig. 7 illustrates this logic on result screens exported from the application itself.

**Fig. 7:**
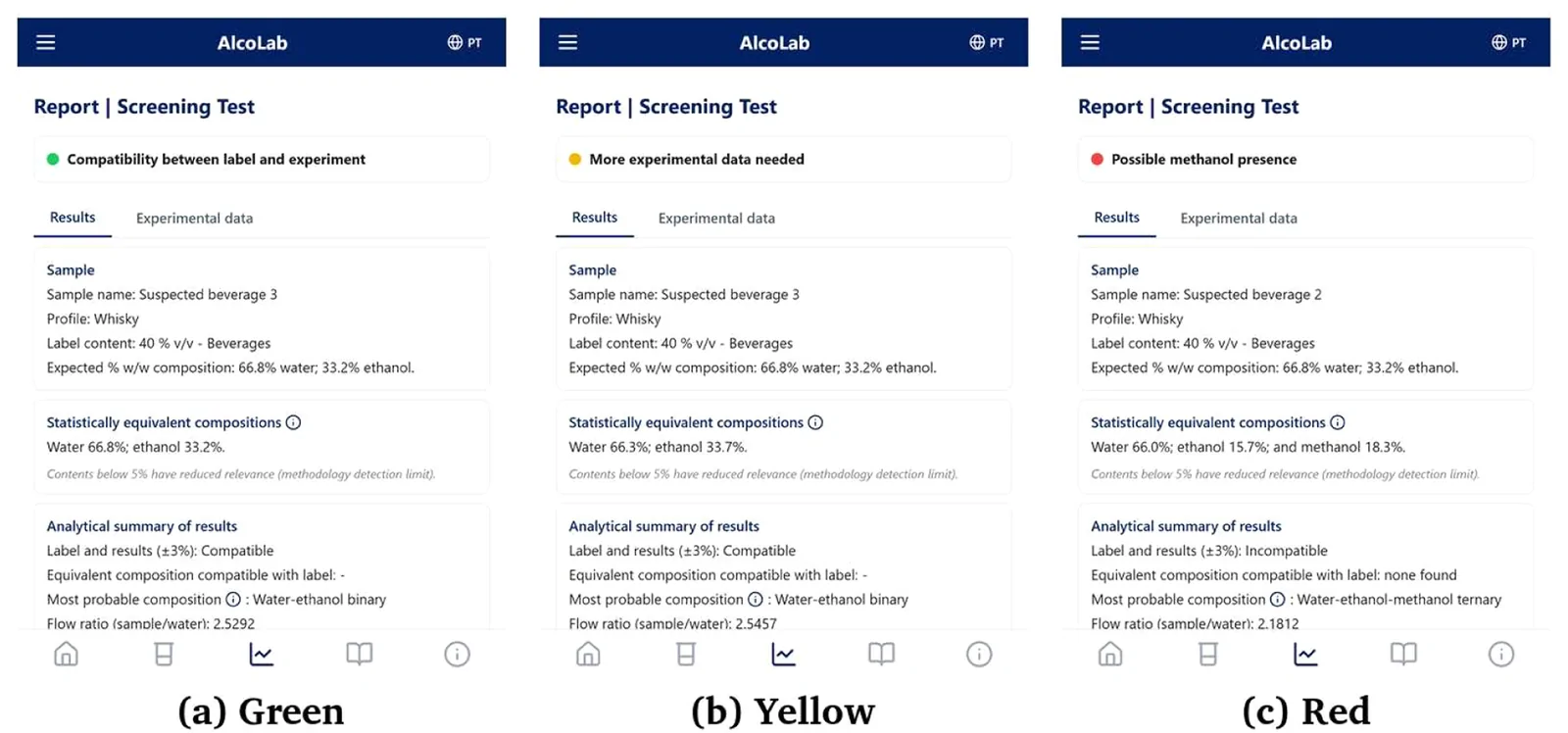
AlcoLab’s traffic-light logic in application result screens.

### Architecture, open source, and reproducibility

AlcoLab was implemented as an open-source progressive web app (PWA) with local, in-browser processing. This architecture lets the calculation workflow run on the user’s own device, which reduces server costs, preserves the privacy of the sample data, and enables offline use after the initial load.

The computational core was written in Python and runs in the browser through Pyodide, a Python distribution compiled to WebAssembly [43]. The same code used in the methodological studies can therefore be embedded in the application, reducing the risk of divergence between the implementation described in this article and the one executed by the user. To keep the interface responsive, processing runs in a Web Worker, separate from the main interface thread.

Reproducibility was reinforced by several implementation decisions, including a fixed random seed for the Monte Carlo simulations, so that the same input yields the same statistical result on different devices. The source code is publicly available on GitHub (github.com/diegoanapolis/alcolab) under the GNU AGPL-3.0 license, and the application can be accessed at alcolab.org. The exact version used in the experiments is identified by the release tag v1.0.0, archived on Zenodo (DOI 10.5281/zenodo.20573733).

The rerunnable scripts of the methodological studies—covering extraction and window screening, fitting of the empirical model, blocked validation, construction and assessment of the viscosity grid, and evaluation of the statistical layer—accompany this work as supplementary material in a reproducible package. A snapshot of this version was deposited in Zenodo with a persistent DOI (10.5281/zenodo.20574038), ensuring the preservation and permanent citation of the analyzed implementation. The AlcoLab software is registered with the Brazilian National Institute of Industrial Property (INPI, computer program registration No. BR 51 2026 001483-2).

## Method validation

### Assessment of the ternary viscosity grid

Three datasets were used, with distinct and clearly separated roles. The ternary interior was *fitted* to the ten points of [35] at 20°C. The final formulation was *selected* among 23 candidates by minimizing the error over the 46 points of [36] at 20 and 30°C, yielding MAE ≈ 0.043 mPa·s and MAPE ≈ 2.47%. Because that same dataset drove the selection, this figure is a model-selection benchmark and not an untouched external validation. The digitization check, the unit standardization, and the curation criterion for inconsistent points are documented in the supplementary material, which also reports how including the excluded points affects the metric.

To quantify the optimism introduced by selecting on the evaluation set, we performed a nested cross-validation of the selection procedure. Because the candidates are fitted on [35] alone, the candidate set is identical across partitions and the outer loop isolates the selection step exactly: in each partition the candidate with the lowest error on the retained portion of [36] was selected and then evaluated on the held-out portion. Under leave-one-out (46 partitions) and under stratified 5-fold and 10-fold schemes repeated 20 times each, the same candidate was selected in 100% of partitions and no increase in error was observed under within-dataset resampling (optimism ≈ 0.00 percentage points). This supports selection stability within this dataset; it does not assess between-study generalization, misspecification of the candidate family, or systematic bias of the dataset itself. Within that limit the reported 2.47% carries no detectable selection optimism, which reflects the fact that the selected formulation outperforms the next distinct candidate by a wide margin (2.47% versus 2.97%).

As a check independent of both fitting and selection, the grid was compared with equilibrium molecular-dynamics predictions of the shear viscosity of the ternary water + methanol + ethanol system at 298.15 K and 1 bar (Green–Kubo formalism, TIP4P/2005 water model) reported by [39], a dataset used neither for fitting nor for selection: MAE ≈ 0.096 mPa·s and MAPE ≈ 6.80% over 7 ternary points. On this held-out set the selected formulation outperformed both the canonical correction (MAPE ≈ 11.9%) and the purely log-geometric limit (≈ 9.3%), indicating that the improvement is not an artifact of selection. These are simulated rather than measured values; the same source also reports the binary subsystems, for which the simulated viscosities deviate from experimental literature values by 4.8% on average (mean absolute deviation over 23 points at 298.15 K). The grid–simulation discrepancy is therefore of the same order as the grid–simulation discrepancy of the reference itself, and this comparison is reported as a plausibility check independent of fitting and selection rather than as an experimental validation.

Beyond these assessments, the interpolation in lnμ (positivity) and the factor $z_{x}\left(1-z_{x}\right)$ (exact preservation of the edges) support the grid despite the scarcity of direct ternary data. All datasets, candidate rankings, and verification scripts are provided in the supplementary material; the roles and metrics are summarized in Table 2.

### Retrospective within-stratum precision

Precision between repeated measurements was assessed retrospectively on the existing replicates, without additional experiments. Strata were defined by composition, operator, hardware configuration and date, which is the same pairing rule the analysis pipeline uses to assign a session water reference. Thirteen strata contained at least two replicates, covering 37 of the 54 eligible measurements.

Across those strata the coefficient of variation (CV) of the sample-to-water flow ratio $A_{\text{obs}}$ had a median of 3.65%, a 90th percentile of 9.54% and a range of 0.42–13.97%. Because every replicate within a stratum shares a single median water reference, $\text{CV}(A_{\text{obs}})$ is mathematically identical to the coefficient of variation of the sample flow time itself; the two are reported as one quantity rather than as independent results. Table 5 breaks these values down by matrix category; the stratum-resolved values were calculated from the deposited per-replicate table using the stratum definition given above.

**Table 5:** Retrospective within-stratum precision of the sample-to-water flow ratio $A_{\text{obs}}$, by matrix category. Strata were defined by composition, operator, hardware and date, following the water-reference pairing rule used by the analysis pipeline; only strata with at least two replicates are included.

Matrix category	Strata	Replicates	Median CV (%)	Range (%)
Distilled spirits (whisky, vodka, cachaça)	6	21	3.89	0.42–6.21
Commercial ethanol (54 °GL)	3	8	3.47	1.43–3.51
Prepared mixtures and reagents	4	8	7.05	3.36–13.97
All strata	13	37	3.65	0.42–13.97

Descriptively, the observed within-stratum CVs were not larger in the commercial matrices. The six distilled-spirit strata gave a median CV of 3.89% with a maximum of 6.21%, and the three commercial-ethanol strata a median of 3.47% with a maximum of 3.51%, whereas the four strata of prepared mixtures and reagents gave a median of 7.05% and contained the two largest values of the whole set (10.38% and 13.97%, both from two-replicate strata of pure reagents). Within this dataset, therefore, the dispersion between repeated measurements in real beverages was comparable to or smaller than that observed in the idealized preparations.

This analysis is descriptive and retrospective. It is conditional on a shared water reference within each stratum, rests on few replicates per stratum, and does not constitute a variance-components study: because operator, hardware, site and session temperature are partially confounded, the strata cannot be used to attribute variability to any single factor. The coefficient of variation between 1 mL intervals within a single video, and the $R^{2}$ of the volume–time regression, describe the quality of an individual acquisition and are not substitutes for precision between measurements.

### Conditional blocked evaluation of the empirical transfer model

The conditional robustness of the empirical model was assessed by blocked evaluation. In each scheme, predictions were generated from fits in which the corresponding composition, hardware configuration, or operator had been held out. This allowed model performance to be evaluated under conditions not included in the calibration set.

#### Scope of this evaluation

In every scheme the compositional search was restricted to a ±2 percentage-point window centered on the *nominal* total alcohol content of each prepared mixture, rather than on an alcohol content estimated from a measured relative density. The results below therefore characterize the flow-measurement, transfer-model and inversion stages *conditional on the total alcohol content being correctly located within that window*; the density stage, and therefore the complete balance-to-result chain, is outside the scope of this evaluation and is addressed under Future directions in the Limitations section. The evaluation is accordingly termed *conditional*. The error of the total alcohol content itself (MAE ≈ 0.013) is bounded by the window width and is reported for completeness rather than as a performance figure.

#### Results

The mean absolute error (MAE) of the mass fraction under the conditional blocked evaluation was ≈0.026 for methanol and ≈0.029 for ethanol, and the 90th percentile (P90) of the methanol error was 0.071. Of the 54 eligible replicates, 26 contained methanol and 28 did not; all 26 methanol-containing replicates were flagged at the 2% cut-off on the predicted methanol mass fraction, and 22 of 84 negative predictions were flagged incorrectly, giving an aggregate false-positive rate of 26%. Per scheme, the false-positive rate was 10/28 (35.7%) when composition was held out, 7/28 (25.0%) for hardware and 5/28 (17.9%) for operator. The 54 unique video replicates generated 160 scheme-specific predictions across the three blocked evaluations; metrics are therefore reported separately by scheme as well as in aggregate.

#### Concentration range of the positives

The 2% figure is the threshold applied to the predicted methanol mass fraction in order to classify a prediction as positive—unrelated to the ±2 percentage-point search window on total alcohol described above—and not a demonstrated detection limit. Every methanol-containing replicate was flagged in every applicable blocked scheme. At the lowest measured methanol mass fraction (0.166, corresponding to a methanol fraction within the alcohol of 0.387), the six underlying video replicates generated 18 blocked predictions, all of which were flagged (18/18). Separately, two exploratory low-content water–ethanol binaries (7.3% and 9.4% ethanol, evaluated without video) showed that the flow response is not saturated at low total alcohol content. Because no ternary mixture with methanol below 0.166 was measured, the operational screening floor for methanol was not characterized; establishing it requires ternary mixtures with low methanol fractions. Fig. 8 summarizes the calibration and the conditional blocked evaluation.

**Fig. 8:**
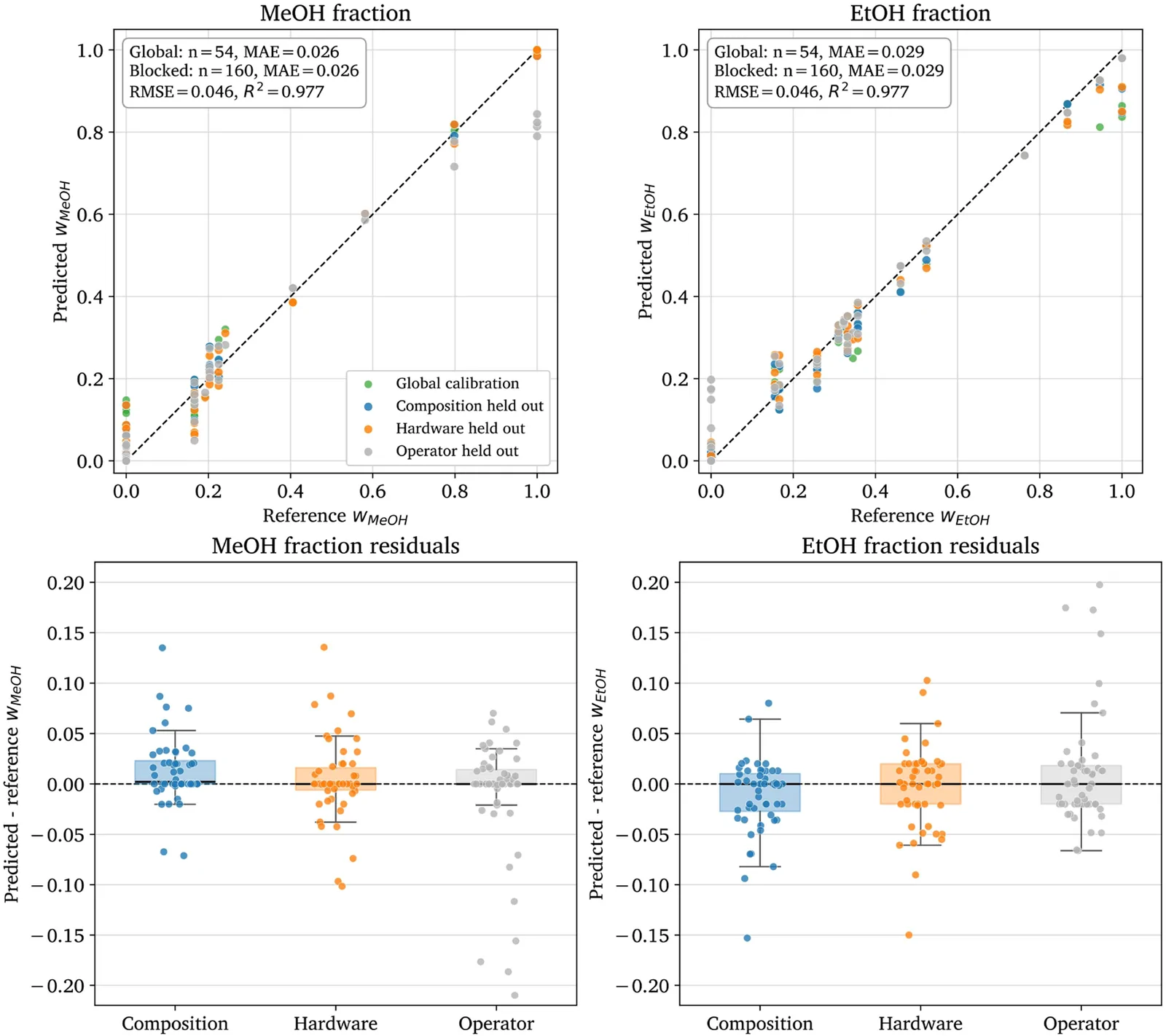
Calibration and conditional blocked evaluation of the empirical transfer model. Parity panels compare predicted and reference mass fractions of methanol and ethanol; the box plots show the corresponding residuals by blocking scheme. The 54 unique video replicates generated 160 scheme-specific predictions. The reported RMSE and $R^{2}$ refer to the blocked set.

#### Design of the blocking factors

Operator, syringe–needle configuration, site and session temperature do not vary independently in this dataset: each operator worked predominantly with one hardware configuration at one site, and one of the operator blocks contains only methanol-free replicates. The blocked schemes are therefore stress tests under combined domain shifts rather than isolated tests of a single factor, and they should not be read as demonstrating independent transferability across operators, sites or hardware.

The largest residuals are concentrated at the near-pure composition vertices of the grid—in particular pure methanol ($w_{\text{MeOH}}\to 1$)—under the schemes that hold out the operator or the hardware. This behavior has two origins: in this proof of concept, the pure-component points were measured by a single operator and with a limited hardware configuration, so holding them out in the blocked evaluation forces the model to extrapolate; and the empirical transfer function is least constrained at the edges of the composition. A practical qualification is nonetheless warranted: this near-pure methanol region is not expected in the real samples targeted by this method. Distilled spirits lie around 30–50% total alcohol and, even in adulteration episodes with toxicologically relevant levels, methanol appears as a fraction of the alcohol—not as a near-pure matrix (for example, 14.6–45.1% in the São Paulo cases). The region at the lower end of the evaluated positive range—intermediate alcohol content, with ethanol and methanol mixed—is also the best sampled and the most constrained by the model, where the sensitivity for flagging methanol was 100%.

### Choice of the viscosity-grid query layer and thermal robustness

The fixed 24°C query layer and the absence of thermal normalization resulted from a post-hoc exploratory analysis on the same blocked-evaluation set. Compared with the previously considered thermal normalization, querying the raw ratio at a fixed layer reduced the methanol and ethanol MAE and the false-positive rate while keeping sensitivity at 100% (methanol ΔMAE ≈ −0.017; 95% confidence interval excluding zero across the whole 18–33°C layer range). With the candidate layers defined a priori by the working range of the sessions (20–24°C), layers from 21 to 26°C were statistically indistinguishable (plateau), so the choice of 24°C—the lowest-error layer—is robust: any layer within the working range would yield equivalent performance. Because this is a selection on the same set, these results are reported as exploratory and motivate an independent confirmation campaign (see Future directions, in Limitations). A complementary structural analysis (grid only) indicates that, with the fixed 24°C layer, the structural thermal deviation remains comparable to the method noise for session temperatures from about 20 to 28°C, degrading above that; this range is adopted as the application’s recommended operating range.

### Retrospective characterization of the plausible set of the statistical layer

This analysis retrospectively characterizes the conservative behavior of the statistical layer in conditional blocked predictions. Because the rule and its thresholds were established during method development using the same experimental dataset, the results are an internal characterization rather than external validation. Unlike the conditional blocked evaluation of the empirical model, which measures the error of the point estimate, this analysis asks whether the reference composition remains represented among the plausible alternatives that the application reports to the user. Over the same set, two quantities were evaluated: (i) the coverage of the compositional families, defined as the fraction of samples of each true family in which that family appeared among the reported alternatives; and (ii) the component-wise minimum absolute error among candidates retained in the plausible set.

Fig. 9a compares the point estimate as evaluated within the plausible-set analysis (MAE ≈0.024 for the methanol mass fraction and ≈0.028 for ethanol) with the component-wise reference-informed minimum among retained candidates (≈0.012 and ≈0.017), whereas the total-alcohol-content error changes little, as expected, since it is already constrained by the density window. Because the minima are taken separately for each component, the candidate that minimizes the methanol error need not be the same one that minimizes the ethanol or total-alcohol error. The metric is retrospective and should be interpreted cautiously: the best candidate can only be identified once the reference composition is known, so it does not replace the evaluation of the point estimate; it merely shows that, when the point estimate is not the closest to the real composition, the conservative set produced by the statistical layer often retains a more suitable alternative.

**Fig. 9:**
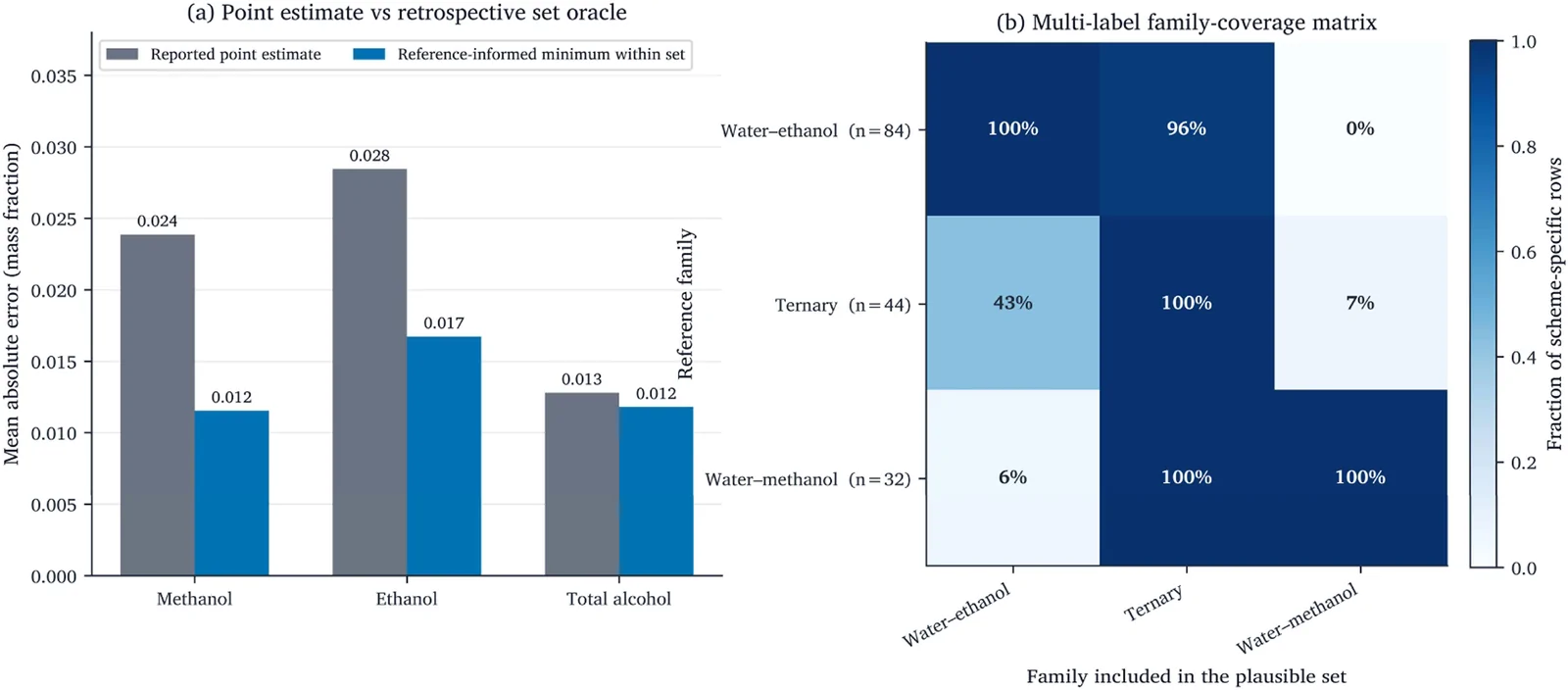
Plausible-set characterization in conditional blocked predictions. (a) Comparison between the reported point estimate and the reference-informed minimum error among members of the plausible set; the latter is retrospective and is not deployable performance. (b) Multi-label family-coverage matrix showing the fraction of scheme-specific predictions in which each family was included. One prediction may include more than one family, so rows may sum to more than 100%.

Fig. 9b shows the coverage of the compositional families. A diagonal of 1.00 indicates that the correct family was present in 100% of the plausible options reported by the application. In this multi-label family-coverage matrix a single prediction may contain more than one compositional family; consequently, the entries in a row are not mutually exclusive and may sum to more than 100%. The off-diagonal cells quantify the expected residual ambiguities: 43% of the ternary samples were also compatible with the water–ethanol family and 7% with the water–methanol family, reflecting regions of the grid where distinct compositions give close viscometric responses within the experimental uncertainty. Together, the two analyses suggest that the statistical layer is conservative while retaining discriminatory value: it does not overstate the model’s predictive capacity but communicates uncertainty responsibly, pairing the point estimate with plausible alternatives and preventing a single composition from masking the physicochemical ambiguity of the ternary inversion.

## Limitations

The method implemented in AlcoLab is presented as a proof of concept, with its limitations stated transparently. The experimental stage was carried out without dedicated funding and with limited laboratory infrastructure, which constrained the breadth of the calibration and evaluation performed at this stage. The method performed consistently across the conditions evaluated, and the application is already available for use and testing by third parties; broader validation studies, with adequate infrastructure and resources, will help map the method’s sensitivity and operational limits more precisely in wider application scenarios.

Regarding the range of methanol concentrations tested, every methanol-containing sample in the video calibration and validation set has methanol as a substantial fraction of the alcohol. Across the 26 methanol-containing replicates the lowest value was z=0.387—that is, 38.7% of the total alcohol mass—and the lowest methanol mass fraction was 0.166. To probe the low-concentration regime, three of the samples assayed are reported below, with three repetitions each, measured only by manual timing, without video capture; for this reason they are not part of the blocked validation and are treated as exploratory evidence (Table 6).

**Table 6:** Additional exploratory samples, in diluted regimes or outside the video-evaluated set, evaluated only by manual timing (without video capture), with three repetitions each. Mean flow time of the reference water = 111.3 s. Mass fractions relative to the total sample mass; error in percentage points (pp).

Sample	n	$t_{\text{sample}}$ (s)	Prepared EtOH/MeOH (%)	Estimated EtOH/MeOH (%)	Error EtOH/MeOH (pp)
EtOH 7.3%	3	181.8	7.3/ 0.0	9.3/ 0.0	2.0/ 0.0
EtOH 9.4%	3	162.1	9.4/ 0.0	8.0/ 1.5	2.7/ 1.5
EtOH 7.8% + MeOH 15.3%	3	184.1	7.8/ 15.3	1.6/ 21.5	6.1/ 6.2

These samples do not define a limit of detection for methanol; they explore response regimes outside the main video-evaluated set. As Table 6 shows, the two low-content binaries responded to ethanol without spuriously flagging methanol (at most a 1.5% indication), and the low-ethanol ternary correctly flagged its 15.3% methanol, although overestimated. The detection of low ethanol contents is indirect evidence that the flow response is not saturated at low alcohol fractions, not a direct demonstration of sensitivity to methanol.

### Performance at low methanol fractions has not been established

The 2% value used in the classification is a decision threshold applied to the predicted methanol mass fraction—and is unrelated to the ±2 percentage-point search window on total alcohol—not a demonstrated detection capability at that level; we therefore make no quantitative claim about an operational screening floor. Characterizing one will require dedicated experiments with ternary mixtures containing methanol at low and intermediate fractions; until then, a negative result must not be read as evidence that a sample is free of methanol.

There are also experimental limitations. The performance tests rely on three syringe–needle assemblies—covering two syringe brands and distinct production batches—together with a small number of operators and a narrow temperature range centered on ambient conditions; generalization to other manufacturers and models, although expected for the same specifications, has not yet been demonstrated. The ternary grid, although built from the literature, has its interior fitted to a limited set of ternary points and selected on a second literature dataset, so that no untouched experimental set remains for external validation; the independent check available is against molecular- simulation data, whose own deviation from experiment is of the same order as the discrepancy being measured. A further structural caveat concerns the upper end of the computational grid: above approximately 41°C the water–ethanol and water–methanol edges converge, the edge contrast approaches zero (minimum ≈ 0.001 in lnμ units, against ≈ 0.086 in the 20–30°C range), and any correction expressed as a fraction of that contrast loses physical meaning. This lies outside the declared quantitative validation range (20–30°C) but bounds the reuse of the grid at higher temperatures. No direct paired comparison with a reference method such as gas chromatography was performed at this stage. Finally, all claims are bounded by the applicability domain and the pre-analytical exclusion criteria stated in Method details, which are also displayed on the application’s home page.

Despite its proof-of-concept character, the method is already useful as a screening test to flag samples in which methanol is present as a substantial fraction of the alcohol—precisely the scenario of the documented cases for which quantitative results are available. In the state of São Paulo in 2025, of the roughly 300 bottles analyzed by the Polícia Científica by gas chromatography, about half showed methanol levels between 14.6% and 45.1% [4]; in Iran, during the 2020 poisoning outbreak [44], gas chromatography of seized illegal beverages detected methanol as a contaminant at high fractions, with a mean content of 25.3 g/dL in the positive samples and an estimated methanol/ethanol proportion above 35% [45]. The initial choice to work with higher methanol fractions was deliberate: it sought to reproduce these episodes and to prioritize the levels of greatest toxicological concern.

### Future directions

Future work is organized along two fronts. The first is expanded validation: expanding the experimental database with more hardware, operators, and temperatures, adding ternary mixtures with methanol at low and intermediate fractions, and including a paired comparison with gas chromatography. Because the model architecture separates the physicochemical reference grid from the empirical hardware transfer function, this expansion can proceed incrementally and at low cost. A natural extension is to test a formulation in which the operational flow-time ratio is corrected explicitly by the measured density of the sample, separating the dominant kinematic contribution from the residuals associated with geometry, entrance and exit effects, and video processing. This effort is intended to involve official laboratories and research centers, supported by funding mechanisms that preserve free access to the tool and support its continuous improvement.

The second front is scope expansion: the same physical principle—density constraining the total alcohol content and flow discriminating between the alcohols—applies, in principle, to methanol screening in fuel ethanol, a matrix linked to the origin of the recent Brazilian crisis. Its formal evaluation, with real samples and in coordination with fuel-inspection agencies, is a natural extension of the method.

In summary, AlcoLab shows that useful compositional information can be extracted from a hydroalcoholic mixture using three simple measurements—mass, time, and meniscus position—by combining a viscosity grid built from the literature, a few-parameter empirical transfer model, and a conservative statistical layer. Its main contribution is not a single performance figure but an open, reproducible workflow that makes a preliminary apparent ternary-equivalent estimate of the water, ethanol, and methanol proportions accessible at very low marginal cost and without reagents—information that has generally remained confined to laboratory settings. The method is, and should continue to be presented as, a screening tool: its role is to flag suspected samples and prioritize them for confirmatory analysis, not to replace chromatography. Within this scope, the flow and inversion stages proved feasible, internally consistent, and showed preliminary conditional robustness under the combined, partially confounded domain shifts evaluated here. Publishing the method, the code, the underlying data, and the core analysis scripts openly is intended to let other researchers reproduce, critique, validate, and improve the workflow—a central purpose of a method article.

## Ethics statements

Not applicable. The research did not involve human subjects, animal experiments, or data collected from social media platforms.

## Declaration of Generative AI and AI-assisted technologies in the writing process

The study design, analytical methodology, software requirements, monitored variables, and overall code architecture were defined and specified by the authors. Within this human-defined framework, generative AI-assisted tools (Claude Code and Claude Cowork, by Anthropic, and Codex, by OpenAI) were used to support the implementation of the data-analysis scripts, programmatic figures, and the application’s source code, as well as to improve the language and readability of the manuscript; additional AI models accessed via API were consulted for language checking. All AI-assisted code and text were reviewed, tested, and validated by the authors against the results reported in this work. The authors take full responsibility for the content of the published article.

## CRediT authorship contribution statement

**Diego Mendes de Souza:** Conceptualization, Methodology, Software, Formal analysis, Investigation, Data curation, Validation, Visualization, Writing – original draft, Writing – review & editing. **Pedro Augusto de Oliveira Morais:** Conceptualization, Methodology, Formal analysis, Investigation, Validation, Supervision, Writing – review & editing. **Nayara Ferreira Santos:** Validation, Resources, Project administration, Writing – review & editing. **Romério Rodrigues dos Santos Silva:** Investigation, Validation, Writing – review & editing.

## Declaration of competing interest

The application and its source code are released free of charge as open-source software under the GNU AGPL-3.0 license (registered with the Brazilian INPI, computer program registration No. BR 51 2026 001483-2). After this study and the registration, the company Baru Tech was established and will be responsible for the application’s continuity. The company’s partners are Nayara Ferreira Santos, Diego Mendes de Souza, and Pedro Augusto de Oliveira Morais. The authors declare no other competing financial interests or personal relationships that could have influenced the work reported in this paper.

## Data Availability

Data, videos, frames, grid files and core analysis scripts are available at https://doi.org/10.5281/zenodo.20574038; software at https://doi.org/10.5281/zenodo.20573733.
