# Supplementary material for: AlcoLab: a method for methanol screening using density and syringe-flow measurements, implemented as an open-source application
Source: MethodsX. 2026 Oct 19;17:104211. doi: 10.1016/j.mex.2026.104211 (PMC13735008; doi:10.1016/j.mex.2026.104211)
Supplement: Supplementary file 1 [file mmc1.pdf]

Supplementary Material

AlcoLab: a method for methanol screening using density and syringe-flow measurements, implemented as an open-source application

Diego Mendes de Souza[a], Pedro Augusto de Oliveira Morais[b,*], Nayara Ferreira Santos[a], Romério Rodrigues dos Santos Silva[c]

[a] *Baru Tech, Brasília, DF, Brazil*
[b] *Universidade Federal do Maranhão (UFMA), Centro de Ciências Exatas e Tecnologia (CCET), Campus Bacanga, São Luís, MA, Brazil*
[c] *Universidade Federal do Maranhão (UFMA), Centro de Ciências de Chapadinha, Chapadinha, MA, Brazil*

This document is the guide to the supplementary material of the article. The material itself (experimental data, raw videos and frames, viscosity-grid files, analysis outputs and reproduction scripts) is permanently archived in a public repository, because its total size (48.6 GB, of which 48.4 GB are raw syringe-flow videos) exceeds what can be distributed with the article. Table S2 lists every item referred to as supplementary in the article and the file in which it is found.

1. Deposited records

Table S1. Public records containing the supplementary material and the application source code.

Record	Persistent identifier	Files (size; MD5)
**Supplementary data, videos and scripts.** "AlcoLab — MethodsX supplementary material: data, videos, scripts and reproduction package" (Zenodo; dataset; CC BY 4.0)	`https://doi.org/10.5281/zenodo.20574038`	`alcolab_supplementary_data_code.zip` (1.3 MB; `ab6112ebfd1bbcaafb29fbb9b5296c24`)<br>`frames.zip` (180.9 MB; `14a12fc304919a37aa11e9de3af85fc8`)<br>`videos.zip` (48.4 GB; `8306699aa25cd0f2e6e86c096f076e25`)
**Application source code**, release v1.0.0 used in the experiments (Zenodo; software; GNU AGPL-3.0)	`https://doi.org/10.5281/zenodo.20573733`<br>`https://github.com/diegoanapolis/alcolab` (tag v1.0.0)	`alcolab-v1.0.0.zip` (8.9 MB; `5f7555bda7e2cdb1cd0f17eab879a454`)

Unpacking `alcolab_supplementary_data_code.zip` restores the folders `1_data/`, `2_code/`, `3_videos/` and `4_app/`, together with `README.md` and the integrity manifest `MANIFEST_SHA256.csv`. The 101 videos (`videos.zip`) and the extracted frames (`frames.zip`) unpack into `3_videos/`; per-video SHA-256 checksums are given in `3_videos/video_checksums_sha256.csv`. Figure and table numbers below follow the published article; the `README.md` inside the package was prepared with the numbering of an earlier version of the manuscript.

*Corresponding author.
Email address: `morais.pedro@ufma.br` (Pedro Augusto de Oliveira Morais)

2. Items cited in the article and their location in the deposited package

Table S2. Supplementary items cited in the article and the corresponding files.

**Location in the article**	**Item**	**File(s) in the deposited package**
Highlights; Method details – Architecture, open source, and reproducibility	Rerunnable scripts of the methodological studies and supplementary videos	`2_code/` (all scripts; `reproduce_all.sh`); `videos.zip`; `frames.zip`
Method details – flow-time measurement (18–14 mL window)	Window screening (increase in flow CV below 14 mL)	`1_data/window_screening.csv`; `1_data/marks_and_windows_public.csv`
Method details – Figure 3	Video `AlcoLab_V004` (commercial whisky; hardware SR–SR; 23 °C) and its meniscus marks	`videos.zip`: `AlcoLab_V004_whisky.mp4`; `frames.zip`: `AlcoLab_V004` frames; `1_data/meniscus_marks.csv`
Method details – Ternary grid anchored in the literature; Table 2	Full ranking of the 23 grid candidates; original grid-construction scripts; Song (2008) anchor data	`1_data/ternary_mesh/external_validation_metrics.csv`; `song2008_points.csv`; `song2008_anchor_terms.csv`; `mesh_20_50C_*.npz`; `parameters.yaml`; `2_code/build_mesh.py`
Method details – Empirical transfer model; Table 3	Per-replicate data of the 54 eligible replicates used in the fit	`1_data/master_table.csv`; `1_data/blocked_validation/final_model_per_replicate_predictions_public.csv`
Method details – Empirical transfer model (real commercial matrices)	Matrix identity and spiked/ unspiked condition of each replicate	`1_data/master_table.csv` (columns `composition_group`, `supplement_filename`); `3_videos/video_index.csv`; `1_data/hardware_dict.md`
Method details – Empirical transfer model (same-session water reference)	Calibration of the absolute flow time, $k_{\mathrm{HW}}(T)$ and k_{ideal} (quality control; not used in the inversion)	`2_code/final_parameters.yaml` (quality-control constants); `1_data/water_temperature_20C/`
Method details – Empirical transfer model (exploratory V_{sin} form)	Parameters of the exploratory model evaluated and not retained	`2_code/vsin_exploratory_model.py`; `2_code/reproduce_empirical_model.py`; `2_code/final_parameters.yaml`
Method details – Empirical transfer model; Ternary inversion (Figure 6)	Script `final_model.py`, function `invert_final_model` (Eqs. 3–6)	`2_code/final_model.py`; `2_code/final_parameters.yaml`; `1_data/final_model_parameters.csv`
Method details – Statistical pre-interpretation layer	Reference standard deviation used when only one replicate is available	`2_code/final_parameters.yaml` (`sigma_ref_single_replicate_A`)
Method details – Statistical pre-interpretation layer	Convergence study of the Monte Carlo simulation (N = 3000 against N = 200,000)	`1_data/statistical_layer/mc_convergence.csv`; `2_code/reproduce_statistics.py`
Method validation – Assessment of the ternary viscosity grid	Digitization, unit standardization and curation of the Dizechi & Marschall points; excluded points	`1_data/dizechi_marschall/` (`curated_points.csv`, `excluded_points.csv`, `curation_criterion.md`, `curation_sensitivity_summary.csv`); `2_code/external_mesh_validation.py`
Method validation – Assessment of the ternary viscosity grid	All datasets, candidate rankings and verification scripts	`1_data/`; `2_code/`; smoke check `2_code/check_article_results.py`
Method validation – Retrospective within-stratum precision; Table 5	Deposited per-replicate table from which the stratum values were calculated	`1_data/master_table.csv`

Location in the article	Item	File(s) in the deposited package
Method validation – blocked evaluation and plausible set (Figures 8 and 9)	Blocked-validation results by scheme; plausible-set validation outputs	1_data/blocked_validation/; 1_data/statistical_layer/plausible_set_validation_*.csv

3. Reproduction

All scripts run from the public package alone; the computational environment is given in 2_code/environment.yml. The script reproduce_all.sh runs the smoke check 2_code/check_article_results.py, which verifies the main published numerical results against the shipped result tables and confirms that every indexed video is present. The application itself is available at https://alcolab.org; its source code is cited in Table S1.
